# Squaring the EMC – how promoting membrane protein biogenesis impacts cellular functions and organismal homeostasis

**DOI:** 10.1242/jcs.243519

**Published:** 2020-04-24

**Authors:** Norbert Volkmar, John C. Christianson

**Affiliations:** 1 Jeffrey Cheah Biomedical Centre, Department of Medicine, University of Cambridge, Cambridge Biomedical Campus, Cambridge CB2 0XY, UK; 2 Oxford Centre for Translational Myeloma Research, Nuffield Department of Orthopaedics, Rheumatology and Musculoskeletal Sciences, University of Oxford, Botnar Research Centre, Headington, Oxford OX3 7LD, UK

**Keywords:** EMC, Membrane protein, Protein quality control, Protein folding

## Abstract

Integral membrane proteins play key functional roles at organelles and the plasma membrane, necessitating their efficient and accurate biogenesis to ensure appropriate targeting and activity. The endoplasmic reticulum membrane protein complex (EMC) has recently emerged as an important eukaryotic complex for biogenesis of integral membrane proteins by promoting insertion and stability of atypical and sub-optimal transmembrane domains (TMDs). Although confirmed as a bona fide complex almost a decade ago, light is just now being shed on the mechanism and selectivity underlying the cellular responsibilities of the EMC. In this Review, we revisit the myriad of functions attributed the EMC through the lens of these new mechanistic insights, to address questions of the cellular and organismal roles the EMC has evolved to undertake.

## Abstract (precis)

**Summary:** The EMC is an important factor facilitating membrane protein biogenesis. Here we discuss the broad cellular and organismal responsibilities overseen by client proteins requiring the EMC for maturation.

## Introduction

The eukaryotic endoplasmic reticulum (ER) houses the machinery responsible for biogenesis of secreted and integral membrane proteins. Insertion, folding and maturation of nascent polypeptides is coordinated by a collection of ER-resident factors, many of which remain poorly understood. More than a decade ago, a genome-wide screen monitoring the unfolded-protein response (UPR) identified and characterised factors essential for protein folding and ER homeostasis in yeast (Jonikas et al., 2009). Among these was a cluster of genes, whose deletion exacerbated ER stress and whose gene products co-precipitated as a complex. Based on its organisation and localisation, the hetero-oligomer was designated as the ER membrane protein complex (EMC) (Jonikas et al., 2009). Several years later, the mammalian orthologue was independently identified using mass spectrometry (MS) through links to ER quality control components (Christianson et al., 2012). The EMC is evolutionarily conserved, with origins reaching back to the last eukaryotic common ancestor (LECA) (Wideman, 2015). An ancient heritage reflects strong positive selection throughout evolution to retain the EMC and the cellular function(s) it performs. EMC functionality had remained enigmatic, due in part to the disparate phenotypic outcomes that resulted from targeted disruption of individual EMC subunits. This changed recently with a flurry of studies providing compelling evidence that the EMC can function as an insertase, promoting biogenesis of transmembrane domain (TMD)-containing proteins at the ER. Here, we discuss recent advances in EMC biology and re-evaluate the phenotypes once attributed to the EMC in light of this new functional insight.

## Features of the EMC

**Fig. 1.**
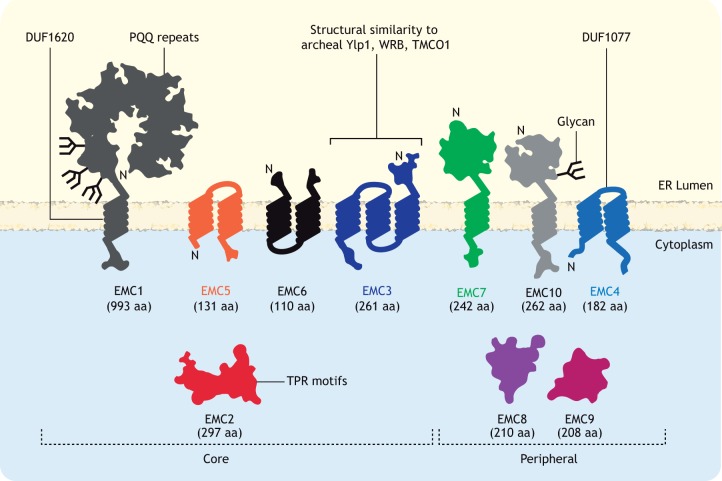
**Schematic overview of the EMC.** The mammalian EMC contains ten subunits (EMC1 to EMC10), with EMC8 and EMC9 being homologues. Bioinformatic analysis of subunit topologies predicted significant mass on either side of the ER membrane, connected by membrane-spanning domains in seven out of ten subunits. At a 1:1 stoichiometry, the EMC is anticipated to contain at least 12 TMDs (Goytain and Quamme, 2008; Guna and Hegde, 2018; Richard et al., 2013; Ring et al., 2008; Smirle et al., 2013). EMC1, EMC7 and EMC10 were predicted to contain signal sequences and adopt a type-I transmembrane protein topology (Junes-Gill et al., 2011; Ninagawa et al., 2015), which was confirmed by the detection of N-linked glycans on both EMC1 and EMC10 (Chen et al., 2009; Junes-Gill et al., 2011; Ninagawa et al., 2015). EMC3, EMC4, EMC5 and EMC6 appear to be polytopic, whereas EMC2, EMC8 and EMC9 have no ER-targeting signals or TMDs, and are presumed to assemble with one or more membrane-integrated EMC subunits at the cytoplasmic interface of the ER. Multiple predicted motifs (TPR motif) and domains (DUF1077, DUF1620, PQQ repeats) are indicated. Depletion of ‘core’ subunits (EMC1, EMC2, EMC3, EMC5, EMC6) destabilises the complex or interferes with EMC assembly, whereas loss of ‘peripheral’ subunits (EMC4, EMC7, EMC8, EMC9, EMC10) has a mild or no discernible effect on EMC expression. aa, amino acids; N, N-terminus.

The EMC was first described as an integral membrane protein complex in yeast, with similar genetic interaction patterns and whose six protein products co-precipitated as a hetero-oligomer (Jonikas et al., 2009). Two other membrane proteins, SOP4 and YDR056C, also co-purified with this complex (Jonikas et al., 2009) and would later be re-classified as EMC7 and EMC10, respectively, to yield a mature complex of at least eight subunits (Wideman, 2015). An MS-based mapping of the ER-associated degradation (ERAD) interaction network not only confirmed the EMC orthologue in mammals but also identified EMC8 and EMC9, two metazoan-specific subunits that share over 40% sequence identity (Christianson et al., 2012). A schematic overview of all mammalian EMC subunits is presented in Fig. 1.

Isolation of the mature EMC under stringent solubilisation conditions indicated robust inter-subunit interactions (Christianson et al., 2012; Jonikas et al., 2009). EMC structural integrity depends wholly or in part on ‘core subunits’ (EMC1, EMC2, EMC3, EMC5, EMC6) that are essential for both assembly and function (Guna et al., 2018; Shurtleff et al., 2018; Volkmar et al., 2019). Disrupting any core subunit leads to co-translational degradation of other subunits, failed assembly of the mature EMC and loss of its activity (Volkmar et al., 2019). By contrast, loss of any of the ‘peripheral’ subunits (EMC4, and EMC7 to EMC10) of the EMC does not seem to notably compromise complex stability or assembly. However, the absence of some peripheral EMC subunits, such as EMC4, can compromise EMC function (discussed below) (Shurtleff et al., 2018; Volkmar et al., 2019). How other peripheral subunits contribute to EMC functionality has yet to be fully appreciated but notable links to disease suggest their contributions are important. Although insight into EMC stoichiometry, topology and structure is limited, multiple lines of evidence indicate that subunits assemble with a stoichiometry of ∼1:1 (Guna et al., 2018; Hein et al., 2015; Jonikas et al., 2009; Lahiri et al., 2014; Li et al., 2014; Shurtleff et al., 2018). All EMC subunits co-sediment, indicating obligate assembly into a complex; however, unassembled subunits – except, possibly, EMC10 – are unstable and constitutively degraded (Volkmar et al., 2019).

### EMC subunit domains and motifs

The EMC has few functionally annotated domains or motifs, a fact that has frustrated investigations into its cellular role(s). Aside from hydrophobic TMDs and signal sequences, only EMC1, EMC2 and EMC3 contain annotated domains. EMC1 is by far the largest subunit and harbours pyrroloquinoline quinone (PQQ)-like repeats (aa 21–252) within the region exposed to the ER lumen and a domain of unknown function (DUF1620, aa 787–992) that encompasses the TMD-containing C-terminus (Ninagawa et al., 2015). PQQ repeats adopt a β-propeller fold – often with six to eight blades – and are typically found in proteins using the cofactor pyrroloquinoline. They can serve as structural scaffolds for protein–protein interactions (Kopec and Lupas, 2013). EMC2 contains tandem tetratricopeptide repeat (TPR) motifs; at least five in mammals and one in yeast. A single TPR motif contains two antiparallel amphipathic alpha helices, and the parallel arrangement of multiple TPR motifs gives rise to a large amphipathic groove that is able to accommodate complementary polypeptides (Blatch and Lässle, 1999). TPR motifs are found in a diverse range of proteins including CHIP and HOP, co-chaperones that mediate binding to the C-termini of Hsp70 and Hsp90, respectively (Ballinger et al., 1999; Scheufler et al., 2000). EMC3 shares structural features conserved among members of the Oxa1/Alb3/YidC superfamily of membrane insertases (Anghel et al., 2017; Borowska et al., 2015). Its presence is consistent with the recently described role of the EMC as a TMD insertase, and could reflect key structural and functional roles for EMC3 (Chitwood et al., 2018; Guna et al., 2018).

## The EMC and membrane protein biogenesis

Integral membrane proteins represent ∼30% of the cellular proteome (Krogh et al., 2001), with as many as 5000 different human proteins requiring insertion into the ER membrane during their biogenesis. Although many share common topologies, they still represent a diverse range of sequences and structures following unique folding pathways to maturity. Accommodating this diversity requires a commensurate ensemble of protein biogenesis factors of overlapping specificity and functional redundancy. With biological function intimately tied to accurate membrane integration, precise and efficient insertion of TMD-containing proteins is imperative and requires the ever watchful guidance of dedicated membrane-resident machinery. The EMC has recently been added to this growing molecular toolset, and a detailed review of the current understanding of the EMC mechanism is available (Chitwood and Hegde, 2019). A brief summary of how the EMC functions as a tail-anchored (TA) protein insertase is provided in Box 1, while its role in the biogenesis of polytopic membrane proteins, particularly those containing signal-anchor peptides (SAPs), is discussed in Box 2.

**Box 1. The EMC as a tail-anchor protein insertase**

Tail-anchored (TA) proteins contain TMDs typically located within ∼30 aa of the C-terminus, which are completely shielded by the ribosome exit tunnel until release of the full-length protein, preventing recognition by the signal recognition particles (SRPs) and Sec61-mediated insertion. This scenario necessitates insertion of the TA by a post-translational mechanism.

Cytosolic ‘pre-targeting complexes’ shield TMDs from aqueous surroundings, enabling TA protein transfer to specific targeting factors for delivery and handover to membrane-embedded insertases (reviewed in Guna and Hegde, 2018). TA hydrophobicity varies considerably and, consequently, multiple pathways are required to maintain solubility to ensure accurate targeting and to catalyse post-translational insertion into the ER membrane. Tail-anchor TMDs with high hydrophobic indices preferentially engage the TRC40/GET pathway (Schuldiner et al., 2008; Stefanovic and Hegde, 2007), whereas TA proteins with weak hydrophobic or amphipathic TM helices are poor clients of this pathway. Originally posited to enter through ‘spontaneous’ TA insertion (Brambillasca et al., 2005), recent evidence demonstrated that, for many TRC40-independent TA proteins, the EMC functions as the complementary ER membrane insertase (Guna et al., 2018).

Insight came from a combined approach of proteomics-based discovery and *in vitro* reconstitution of TMD insertion. Investigation of the weakly hydrophobic TMD of squalene synthase (SQS) found that endogenous SQS from EMC-deficient cells degraded rapidly; moreover, recombinant SQS inserted inefficiently into microsomes isolated from those cells and formed cytosolic aggregates when overexpressed (Guna et al., 2018; Volkmar et al., 2019). Insertion of SQS – but not of the TRC40 client VAMP – was reconstituted *in vitro* by using purified EMC embedded in liposomes, which demonstrated both necessity and sufficiency for the EMC to insert weak to moderately hydrophobic TA clients. Biochemical and *in silico* sequence analysis further suggested an evolutionary link between EMC3 and the Oxa1 insertase family that includes Alb3, Get1, TMCO and the bacterial transporter YidC (Anghel et al., 2017; Borowska et al., 2015). It should be noted that Oxa1 family members act as monomers, whereas the EMC assembles as a hetero-oligomer of gene products unrelated to EMC3. It, therefore, would seem that evolution has conscripted other EMC subunits to make important contributions to its function as a TA insertase.

**Box 2. The EMC assists in polytopic membrane protein biogenesis**

For some time, the EMC has been implicated in polytopic membrane protein biogenesis (Louie et al., 2012; Richard et al., 2013; Satoh et al., 2015; Shurtleff et al., 2018; Chitwood et al., 2018; Coelho et al., 2019; Volkmar et al., 2019); a role potentially distinct from that of a TA insertase. The loss of apparently unrelated polytopic membrane proteins producing various phenotypes had coincided with different EMC subunit mutations and deletions. Now with the awareness that EMC function arises from a complex, the current crop of studies is beginning to illuminate how polytopic proteins might be potential clients.

Before the EMC was fully appreciated as an assembled complex, a two-amino acid deletion in EMC3 had been linked to degeneration of red-sensitive opsin-expressing cones in the zebrafish (*D. rerio*) eye (Brockerhoff et al., 1997; Taylor et al., 2005). EMC3 was later shown to be required for biogenesis of the G-protein coupled receptors (GPCRs) rhodopsin 1, 3 and 4 in fruit fly (*D. melanogaster*) photoreceptor cells (Satoh et al., 2015; Xiong et al., 2019). These observations have been put into focus by evidence demonstrating that accurate orientation of signal anchor peptides (SAPs) in select GPCRs requires the EMC, exemplified by the β1 adrenergic receptor (β1AR) (Chitwood et al., 2018). EMC engagement favours SAP sequences with increased length, reduced hydrophobicity and charge ambiguity in cytosolically flanking regions, reminiscent of the preference of EMC for TA segments of low to moderate hydrophobicity (Box 1). Yet, where EMC-mediated TA protein insertion is Sec61-independent, this function – associated with polytopic client proteins – appears to blend in seamlessly with the SRP pathway. Under this paradigm, the EMC co-translationally inserts SAPs to correctly orient the N-terminus within the ER lumen, with Sec61 then integrating any subsequent TMDs. Crucially, β1AR biosynthesis was not quantitatively impaired in rough microsomes isolated from EMC-deficient cells, but approximately half of β1AR molecules adopted an incorrect topology. Although the EMC can autonomously mediate SAP insertion, Sec61 can compensate for its absence but with reduced accuracy. Because correct SAP insertion dictates orientation of ensuing TMDs, the EMC effectively improves topological accuracy of GPCR biogenesis. Without it, topogenesis becomes stochastic and can give rise to inverted forms that are non-functional and potential targets for ERAD.

### EMC-dependent clients

The current spectrum of validated EMC clients is limited but includes both TA and SAP-containing proteins (Table 1). Yet, many of the proteins that appear to be regulated by the EMC do not conform to TA or SAP modalities, which suggests a more general role in TMD insertion and a client range that could eventually reach a significant fraction of the membrane proteome (see also Box 2). Whole-cell proteomic analyses found 26 different polytopic proteins that are significantly depleted in EMC2- or EMC4-deficient cells, even though translation rates remained effectively unchanged (Shurtleff et al., 2018). In yeast, profiles of translating ribosomes proximal to EMC5 revealed enrichment of polytopic membrane proteins with ‘atypical’ TMDs that contain charged residues, including a number of members belonging to the solute carrier (SLC) family of membrane transport proteins, such as SLC43A3 (Shurtleff et al., 2018; Tian et al., 2019).

**Table 1.**
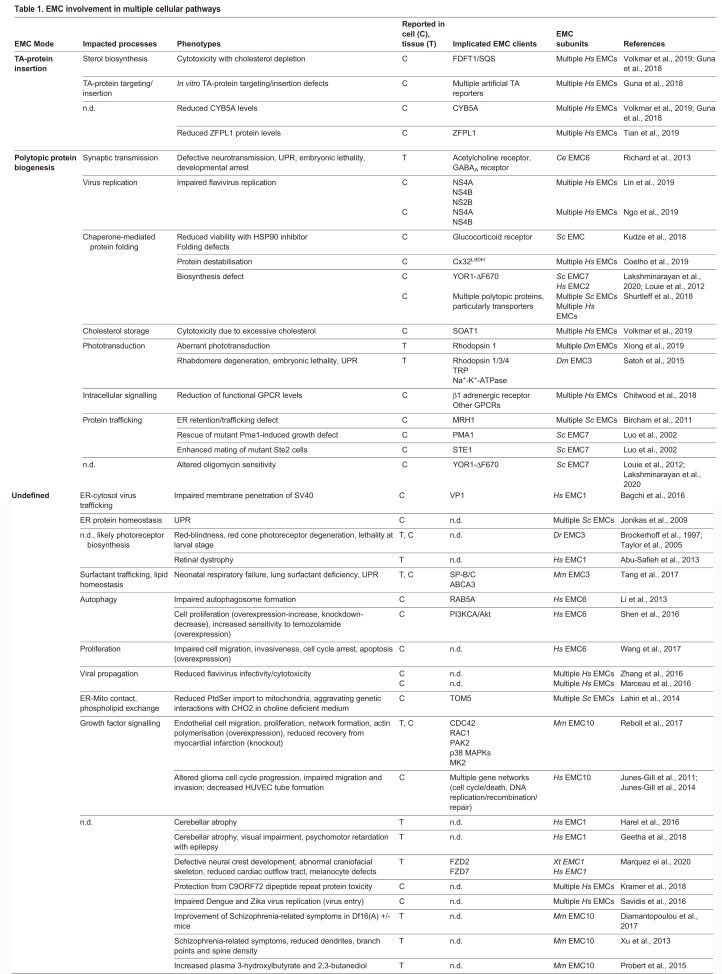
**EMC involvement in multiple cellular pathways**

Quantitative proteomic methodologies have been powerful predictors of EMC-dependent client proteins by monitoring changes in their abundance within EMC-deficient cells (Shurtleff et al., 2018; Volkmar et al., 2019). One limitation may arise from underestimating the number of client proteins that are inserted irregularly but are not degraded, as is evident for some GPCRs (Chitwood et al., 2018). Moreover, functional redundancy with the EMC might also reduce detection of ‘part-time’ clients, whose maturation can also occur via other pathways. This highlights the importance to employ alternative strategies in order to discover clients, such as synthetic lethality screens that use small molecule inhibitors or CRISPR/Cas9, assays that monitor subcellular localisation or those that discriminate between protein topology (e.g. glycosylation, protease protection).

Recognising the origins of phenotypic changes has, in some cases, enabled the responsible EMC clients to be deduced. Defects in synaptic transmission in *Caenorhabditis elegans* were traced back to defective maturation of a nicotinic acetylcholine receptor (Richard et al., 2013), a member of the cysteine-loop class of ionotropic receptors that includes GABA_A_, serotonin, and glycine receptors (Lester et al., 2004). Cysteine-loop, ligand-gated ion channels (LGICs) contain amphipathic helices that, upon oligomerisation, form cationic pores which span the lipid bilayer. Sterol-O-acyltransferase 1 (SOAT1) was uncovered by recognising that surplus cholesterol was synthetically lethal when cells lacked the EMC (Volkmar et al., 2019). SOAT1 esterifies free cholesterol and is a member of the membrane-bound O-acyltransferase (MBOAT) superfamily, for which the first structure of a bacterial orthologue revealed a complex mesh of overlapping and tilted TMDs (Ma et al., 2018). LGICs and SOAT1 do not contain TAs or SAPs but appear to have features within their membrane architecture that makes them difficult to insert and fold properly. Although just beginning to be defined, these features fit well within the proposed model of TMD selectivity by the EMC. Defining the complete cellular repertoire of clients will help to understand why evolution has faithfully conserved the EMC.

In summary, the newly recognised preference of the EMC for a subset of TMDs with general physicochemical features provides a unifying explanation for the dual functionality of the EMC, acting both post-translationally as an insertase for TA proteins and co-translationally as a chaperone/guide for polytopic membrane proteins.

## The EMC as a quality control hub

**Fig. 2.**
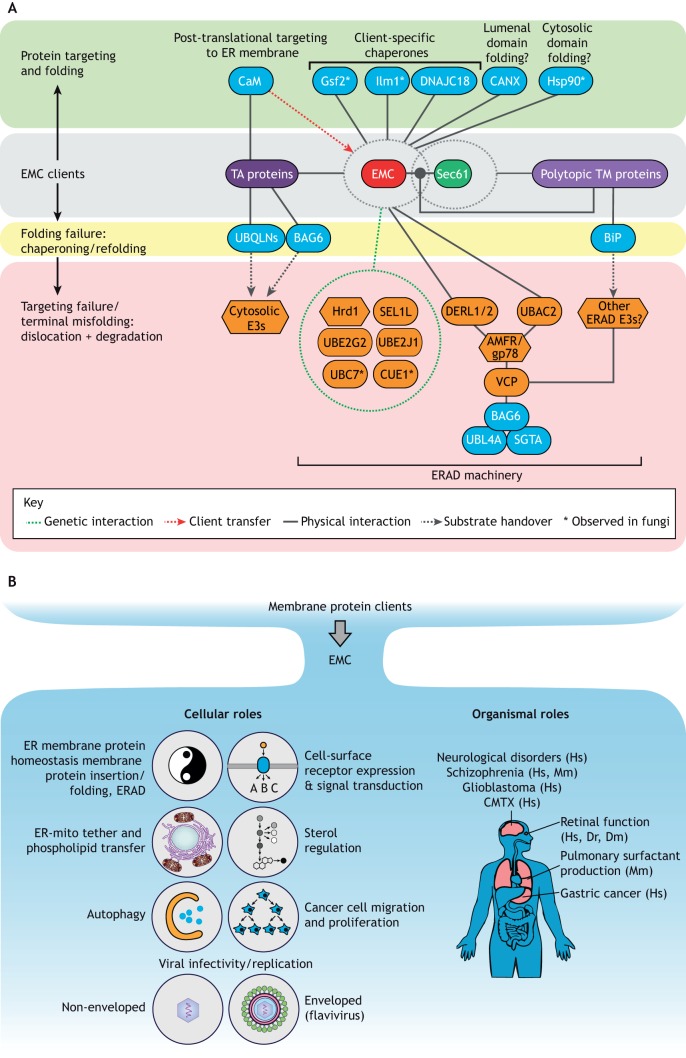
**Client processing by the EMC and** **its consequences.** (A) Triaging between the EMC, Sec61, chaperones and the ERAD machinery. The EMC assists in insertion and folding of membrane proteins within the ER. Although the EMC alone is sufficient for ER membrane insertion of certain tail-anchored (TA) proteins, polytopic proteins are inserted in cooperation with Sec61 (grey background). To fulfil these functions, the EMC–Sec61 module is thought to interact with a variety of targeting and/or folding factors that further assist in membrane protein folding (on green background) and remove terminally misfolded proteins via ERAD or by targeting to cytosolic E3 ubiquitin ligases (on yellow and red backgrounds). Solid lines represent experimentally demonstrated protein–protein interactions. Dashed lines, arrows and circles imply functional links to chaperones or degradation machinery in need of further investigation. Dashed green line and circle indicate genetic interactions. BAG6, large proline-rich protein BAG6; CaM, calmodulin, UBL4A, ubiquitin-like protein 4A; UBQLNs, ubiquilins; VCP, valosin-containing protein. (B) Cellular and organismal roles of the EMC. The EMC exerts a multitude of cellular (left) and organismal functions (right) through its direct or indirect effects on membrane proteins (see Table 1, Boxes 1 and 2). CMTX, Charcot-Marie-Tooth disease; Dm, *Drosophila melanogaster*; Dr, *Danio rerio*; Hs, *Homo sapiens*; Mm, *Mus musculus*.

Membrane protein biogenesis is highly coordinated, requiring cooperation between protein folding and quality control factors across three distinct cellular environments (ER lumen, lipid bilayer, cytosol) to ensure fidelity (Houck and Cyr, 2012). While providing a route to integrate mono- and/or polytopic proteins into the lipid bilayer, the considerable mass and dual aspect of the EMC make it an ideal candidate to scaffold accessory factors. Such factors could promote folding of complex lumenal or cytoplasmic domains within EMC clients or triage misfolded forms to degradation pathways. Multiple lines of evidence implicate the EMC as a ‘quality control hub’ for membrane proteins, functioning as a context-dependent binding partner for both general and specialised molecular chaperones (Bagchi et al., 2016; Coelho et al., 2019; Kudze et al., 2018; Richard et al., 2013; Shurtleff et al., 2018; summarised in Fig. 2A).

### The EMC and ER lumenal chaperones

Through EMC1, the EMC exhibits considerable exposure to the ER lumen (Fig. 1). The glycoprotein chaperone calnexin (CANX) engages the EMC through EMC1 (Christianson et al., 2012; Coelho et al., 2019; Satoh et al., 2015), while also recognising EMC clients themselves, e.g. rhodopsin-1 and the gap junction beta-1/connexin-32 protein (GJB1, hereafter referred to as Cx32) in humans (Coelho et al., 2019). Because interaction with nascent chains is co-translational (Chen et al., 1995), CANX and the EMC might be envisaged to cooperate in folding of selected polytopic glycoproteins. How cooperation might occur is unclear but, as many EMC clients are likely to be glycosylated, this will be an important interface to investigate. Subunits of cysteine-loop LGICs are strong candidates to benefit from such synergy as their maturation depends on both CANX (Wanamaker and Green, 2007) and the EMC (Richard et al., 2013).

The ER-resident Hsp70 family A member 5 (HSPA5, hereafter referred to as BiP) also appears to work together with the EMC to deal with improperly inserted clients. Mutation of TMDs in Cx32 is associated with Charcot-Marie-Tooth disease, and leads to binding of these mutants by BiP, followed by ubiquitylation and degradation instead of correct Cx32 membrane insertion aided by the EMC (Coelho et al., 2019). Moreover, faulty TMD integration into the lipid bilayer and lumenal exposure of hydrophobic domains that result in BiP engagement might predict a link to ER stress (discussed below).

### EMC and ER interactions with membrane-bound chaperones or cofactors

EMC interactions with membrane-embedded chaperones or co-factors represent a second level of synergy. EMC1 binds to DNAJC18 (Bagchi et al., 2016), where the cytosolically oriented J-domain recruits an Hsp70–SGTA–Hsp105 complex to the ER membrane, which is capable of extracting proteins from the lipid bilayer (Goodwin et al., 2011; Ravindran et al., 2015; Walczak et al., 2014). The DNAJC18–EMC1 complex plays an important role in extracting partially destabilised polyomavirus SV40 from the ER membrane (Bagchi et al., 2016). Whether other endogenous or viral client proteins depend on DNAJC18 is unknown but their binding to DNAJ proteins might represent a strategy to clear misfolded client proteins from the EMC. Equally, other membrane embedded DNAJ proteins (e.g. DNAJC16, DNAJC12) might perform comparable activity but for a different repertoire of client proteins.

Before being recognised as part of the yeast EMC (Jonikas et al., 2009), the ER membrane protein complex subunit 7 (EMC7; hereafter referred to as Sop4) had been described as a biogenesis factor involved in maturation and trafficking of the plasma membrane ATPase 1 (Pma1) (Luo et al., 2002). Louie and colleagues, carried out a screen for factors promoting CFTRΔF508 maturation in yeast, and found that Sop4 also clusters together with EMC2 and EMC4 (Louie et al., 2012). Strikingly, the genetic interaction profiles of EMC7 can differ from those of other ‘core’ EMC subunits to such an extent that it can be argued that it serves as a constitutively engaged EMC-associated auxiliary chaperone with client-specific function(s) (Jonikas et al., 2009; Shurtleff et al., 2018). Recently, EMC7 (as well as the EMC) has been linked to TMD handling at early stages of membrane protein synthesis of a misfolded yeast membrane protein that does not contain a TA or SAP (Lakshminarayan et al., 2020). Other substrate-specific chaperones associated with the EMC in yeast include Gsf2 and Ilm1 (Shurtleff et al., 2018); Gsf2 and Ilm1 are involved in the biosynthesis and trafficking of the glucose transporter Hxt1 and the catalytic subunit of 1,3-beta-D-glucan synthase Fks1, respectively (Markovich et al., 2004; Sherwood and Carlson, 1999). Notably, Fks1 was also found to co-precipitate in a complex with the EMC (Shurtleff et al., 2018). This example raises the possibility that the EMC coordinates with client-specific chaperones to enhance accuracy of insertion and stability during biogenesis of key membrane proteins.

### EMC and cytosolic chaperones

EMC2, EMC8 and EMC9 do not contain ER-targeting signals and, thus, form part of the cytoplasmic interface of the EMC. The TPR repeats within EMC2 are likely to participate in protein–protein interactions – but whether those interactions are with subunits, cofactors or client proteins is not yet established. EMC2 has been identified as a putative Hsp90 interactor in multiple genome-wide screens in yeast as it physically interacts with Hsp90 (McClellan et al., 2007; Zhao et al., 2005). Furthermore, in yeast strains that lack the Hsp90 co-chaperone Sti1, loss of EMC2 exacerbates sensitivity to the Hsp90 inhibitor NVP-AUY922 (Kudze et al., 2018). However, as the TPR motif of yeast EMC2 is dispensable for interaction with Hsp90 (Kudze et al., 2018), it is still unclear how recruitment occurs. Intriguingly, EMC-deletion strains also exhibit reduced activity of the cytoplasmic glucocorticoid receptor, a conventional Hsp90 client protein (Chang et al., 1997; Kudze et al., 2018). An Hsp90–EMC interaction in higher eukaryotes has not been reported but to envision the EMC as a co-chaperone for Hsp90 is appealing since it would – together with the Sec61 translocon – provide a robust mechanism to mature membrane proteins with complex cytoplasmic domains. By delivering TA client proteins to the EMC for insertion (Guna et al., 2018), calmodulin (CaM) could also be considered to have ‘chaperone-like’ activity. Nevertheless, how CaM recognises and communicates with the EMC in order to transfer client proteins is not well understood and to delineate this handoff mechanism will require careful reconstitution.

### Triaging proteins between the EMC and ERAD

Spatiotemporal constraints imposed by complex folding programmes complicate biogenesis of polytopic membrane proteins and increase the risk of error. This heightened risk for manufacturing defects means cells must remain poised to deal with aberrant aggregation-prone folding intermediates, stoichiometrically surplus complex subunits and terminally misfolded protein species that may arise during EMC-dependent maturation. In that event, clearance from the ER membrane may be required – but how that might occur is an outstanding question. Enforcement of protein quality control would be expected to be through ER-associated degradation (ERAD), the multistep process responsible for aberrant protein feature recognition, polypeptide removal from the lipid bilayer (retrotranslocation and dislocation), covalent modification by polyubiquitin chains and targeting to 26S proteasomes for degradation (reviewed in Mehrtash and Hochstrasser, 2019; Olzmann et al., 2013; Ruggiano et al., 2014; Vembar and Brodsky, 2008). The interface between protein folding and degradation in the ER is often murky, with many factors serving dual triaging roles. This makes triaging decisions at the EMC–ERAD interface an area of great interest (summarised in Fig. 2A).

An effort to systematically define the protein–protein interaction network of ERAD factors revealed multiple links to individual EMC subunits (Christianson et al., 2012). Mammalian EMC1, EMC2 and EMC3 interact with ubiquitin-associated (UBA) domain containing 2 (UBAC2) and Derlin-2 (DERL2), proteins implicated in ERAD mediated by the E3 ubiquitin ligase AMFR (also known as gp78). It is tempting to envision a gp78-containing cluster poised to target and degrade defective clients purged from the EMC. In the case of the EMC-dependent protein Cx32, a folding-defective point mutant (Cx32^L90H^) used gp78 for ERAD (Coelho et al., 2019). However, since overexpression of the inactive gp78 mutant (gp78^C341/378S^) only conferred partial rescue, other ER-resident ubiquitin ligases (E3s) might be involved. It must also be considered that ERAD factors could be EMC-dependent clients or that unassembled EMC subunits could be ERAD substrates. Both UBAC2 and DERL2 are members of the rhomboid pseudoprotease superfamily with complex polytopic architectures (Greenblatt et al., 2011) and EMC1 degradation is slower in triple-knockout cells of the lectin-like ERAD factors EDEM1, EDEM2 and EDEM3 (Ninagawa et al., 2015).

In yeast cells, unbiased screens also support functional proximity between the EMC and ERAD. The encoding genes for ERAD factors ubiquitin-conjugating enzyme E2 7 (UBC7) and coupling of ubiquitin conjugation to ER degradation protein 1 (CUE1) have strong aggravating genetic interactions with the EMC (Jonikas et al., 2009). A chemical toxicity screen identified EMC subunits (EMC1 to EMC6) and ERAD factors (i.e. UBC7, CUE1, HRD1/3, UBX4, RPN4) as having underlying hypersensitivity to the cytotoxic antifungal compound sr7575 (Raj et al., 2015). Functional associations between the EMC and the human ERAD machinery, i.e. Hrd1 (SYVN1), SEL1L, UBE2J1, UBE2G2, DERL2, were also evident in a genome-wide screen for host factors involved in West Nile virus (WNV) pathogenicity (Ma et al., 2015) (discussed below). Biochemical and physiological implications of a quality control interface between the EMC and ERAD are just beginning to be addressed, but the notion that disruption of pro-maturation or protein clearance factors yields comparable phenotypes, is consistent with a synergistic relationship. More recently, however, compelling evidence for pre-emptive quality control of misfolded EMC clients at the ribosome has emerged, coupled with translational arrest, to reduce a potentially toxic burden (Lakshminarayan et al., 2020). Clearly, the complex clearance mechanisms for misfolded EMC clients will be an area of intense future study.

## Pleiotropic outcomes of EMC disruption reflect broad responsibilities

Loss of the EMC and its subunits manifest as a range of – often unrelated – cellular and organismal defects, reflecting the potentially extensive client range that depends on it for TMD integration (Table 1). Inaccurate insertion leading to degradation or loss-of-function provides a strong rationale for pleiotropy of many origins. As an insertase and/or membrane protein chaperone, the EMC is required for biogenesis of key proteins in lipid homeostasis, signal transduction and, potentially, many other crucial functions (Fig. 2B). Other primary functions have been proposed for the EMC (Lahiri et al., 2014), but these are likely to stem from indirect effects on maturation-dependent clients. Disentangling the primary relationship between the EMC and client proteins from the multifaceted cellular consequences of client disruption, remains a formidable challenge. This is particularly important for complex diseases, such as neurological disorders, as there is evidence that EMC subunits (and the EMC) influence proteins crucial for neural development and maintenance as well as cancer, where EMC subunit expression is linked to tumour suppression (Box 3). The following sections will survey the emerging links between the EMC and functionality at the cellular and organismal levels.

**Box 3. Role of the EMC in human disease**

Neurological disorders

Several neurological syndromes are linked either directly or indirectly to disrupted expression of EMC subunits. Rare EMC1 mutations are linked to severe human neurodegenerative disorders, which present with developmental delay, cerebellar atrophy, scoliosis, hypotonia, psychomotor retardation, epilepsy and craniofacial abnormalities (Abu-Safieh et al., 2013; Geetha et al., 2018; Harel et al., 2016). Accompanied by visual impairment, they are hallmarked by retinal atrophy (Harel et al., 2016) or dystrophy (Abu-Safieh et al., 2013). The pathophysiology is reminiscent of retinal degeneration arising from defective rhodopsin biogenesis (Mendes et al., 2005), a process shown in model organisms to be dependent on other EMC subunits (Satoh et al., 2015; Taylor et al., 2005) (Table 1). Some symptoms might also be explained by congenital neural crest defects in patients carrying pathological EMC1 mutations (Marquez et al., 2020). In a mouse model for the human schizophrenia-related microdeletion 22q11.2, elevated EMC10 levels in the brain contribute directly to morphological and behavioural characteristics of this pathology (Diamantopoulou et al., 2017; Xu et al., 2013). EMC10^−/−^ mice exhibit abnormal locomotor behaviour and an increased anxiety response (Smith et al., 2018). Finally, the inherited peripheral neuropathy X-linked Charcot-Marie-Tooth disease can be caused by mutations in *GJB1*, which encodes Cx32 (Kleopa et al., 2012). *In vitro*, EMC interaction with the pathogenic variant Cx32^L90H^ partially protected it from ERAD mediated by gp78 (Coelho et al., 2019).

Cancer

Altering the expression levels of EMC subunits reportedly modulates tumour growth in several human cancer models. EMC6 knockdown increases glioblastoma cell proliferation, whereas overexpression slows proliferation, inhibits invasiveness and enhances chemotherapeutic sensitivity – all properties consistent with some role in tumour suppression (Shen et al., 2016). EMC10-2 overexpression also inhibits glioma-induced cell cycle progression and invasion (Junes-Gill et al., 2014). EMC6 overexpression improved survival in a glioma xenograft mouse model, attributed to induction of autophagy and PI3K-Akt-mTOR signalling pathway downregulation (Shen et al., 2016). In gastric cancer cells, EMC6 overexpression reduced invasiveness, inducing cell cycle arrest and apoptosis, but not autophagy (Wang et al., 2017). It is unclear how augmented expression of just one EMC subunit elicits these disease phenotypes but it could reflect a rate-limiting element for EMC assembly or, perhaps, a ‘moonlighting’ gain-of-function role. Importantly, complex disease phenotypes are unlikely to arise from a single client and, rather, are attributable to the collective responsibilities of the EMC. Where the EMC and its clients fit in as tumour suppressors or oncogenic drivers remains an area requiring more-detailed studies.

### EMC and ER stress

Protein misfolding in the ER can lead to proteotoxic stress that requires activation of the unfolded protein response (UPR) to restore homeostasis (Travers et al., 2000). Some *in vivo* models of EMC deficiency have reported UPR activation (Richard et al., 2013; Satoh et al., 2015; Tang et al., 2017), indicating that the EMC plays an important role in maintaining ER homeostasis. It is worth noting, however, that several studies did not observe induction of ER stress in response to EMC loss alone (Bircham et al., 2011; Lahiri et al., 2014; Shen et al., 2016). This inconsistency might be attributed to prominent EMC client proteins found in particular tissues or cell types that may show differences in their ability to elicit ER stress. Many of the knockouts and mutants reported previously had not been conditional, making it challenging to determine those client proteins driving the UPR. This lack of clarity underscores the need to better understand what roles the EMC plays during development and for homeostatic maintenance.

### EMC links to cellular and organismal functions

Loss or mutation of core EMC subunits results in embryonic lethality in organismal models. This indicates requirement of the EMC for maturation of key developmentally regulated proteins and hampers investigations into physiological roles of the EMC (Dickinson et al., 2016; Richard et al., 2013; Satoh et al., 2015; Tang et al., 2017). Yet, conditional and tissue-specific EMC subunit knockouts, as well as depletion of ‘peripheral’ subunits (i.e. EMC10), have enabled the compilation of a context-dependent view of EMC influence. Conditional deletion of EMC3 in murine lung epithelial cells results in catastrophic respiratory failure and death of new-born pups (Tang et al., 2017). This was attributed to the collective disruption of the pulmonary surfactant system on three interconnected levels: (i) faulty proteolytic processing and trafficking of surfactant proteins B and C (SP-B and SP-C, respectively), (ii) aberrant surfactant lipid synthesis and, (iii) induction of the UPR (Tang et al., 2017). SP-B and SP-C are essential for distribution of alveolar surface lipids, and maintenance of low surface tension at the alveolar lipid-air interface (Sunde et al., 2017); but they do not contain obvious TMDs that might engage the EMC. The ATP-binding cassette sub-family A member 3 (ABCA3), which was also reduced in alveolar EMC3-knockout cells, fulfils an important role in surfactant lipid transport and SP trafficking (Fitzgerald et al., 2007). Its polytopic architecture could depend directly on the EMC for maturation but this has not been experimentally confirmed. Despite altered lipid profiles and additional biogenesis defects, remarkably, lung morphogenesis, growth and differentiation were largely unaffected by EMC3 loss. This observation is consistent with perturbation of select client(s) linked to lipid homeostasis. As this tissue-specific approach exemplifies, EMC disruption must be appreciated in terms of multifactorial consequences.

Loss-of-function mutations within the *EMC3* gene correlate closely with degeneration of red-sensitive opsin-expressing cones in zebrafish retina and rhabdomeres in fruit fly (Brockerhoff et al., 1997; Satoh et al., 2015; Taylor et al., 2005). Rhabdomere degeneration was accompanied by UPR activation, and loss of the GPCRs rhodopsin 1, 3 and 4 in photoreceptor cells of EMC3 hypomorphs. EMC3-knockout mice also exhibit loss of rhodopsin (Xiong et al., 2019). Interestingly in flies, export of rhodopsin 1 (Rh1) from the ER requires the cofactor XPORT-A for proper trafficking (Chen et al., 2015). XPORT-A is a TA protein whose TMD properties suggest it is a suitable client for insertion by EMC and, if inefficient insertion of XPORT-A would also indirectly contribute to loss of Rh1, this would further illustrate the potential difficulty in identifying EMC clients underlying complex phenotypes.

A mutagenesis screen in *C. elegans* identified a mutation in EMC6, which desensitised the response to the cholinergic agonist levamisole (Richard et al., 2013) and was subsequently traced back to show impaired maturation of the heteromeric acetylcholine receptor at the neuromuscular junction. The pharmacological selectivity of levamisole combined with a robust physiological output permitted this EMC client to be deduced more easily. Extension of these findings to other cysteine-loop LGICs with similar features and properties enabled other EMC clients to be found (Richard et al., 2013). Thus, considering EMC disruption in the context of specific organs and cell types may be a useful tool to identify the key clients and resultant evolutionary pressure.

### The EMC and phospholipid metabolism

The ER is a main site for biosynthesis and regulation of cellular lipids (Fagone and Jackowski, 2009). Perturbations in lipid-associated proteins and cellular lipids have coincided with EMC disruption (Tang et al., 2017; Volkmar et al., 2019). These changes are likely to be secondary consequences and cell-line specific, and evoke homeostatic responses that produce complex metabolic phenotypes, all of which confounded identifying the origin(s) of their EMC dependence. For example, alveolar cells from EMC3-deficienct mice are depleted of enzymes involved in lipid metabolism, exhibit altered whole-cell lipid profiles and an induction of the UPR (Tang et al., 2017). Phosphatidylcholine and phosphatidylglycerol species were markedly reduced, whereas triglycerides were enriched, and transcriptional suppression of key lipid-associated genes accompanied metabolic changes. Notably, this study reported suppression of 3-hydroxybutyrate dehydrogenase type 2 (BDH2), which converts 3-hydroxybutyrate to acetoacetate (Tang et al., 2017). However, whether this is mechanistically related to the elevated 3-hydroxybutyrate serum levels in EMC10-knockout mice (Probert et al., 2015) remains to be confirmed.

Increases in free fatty acids (Cunha et al., 2008), cholesterol (Feng et al., 2003) and levels of ceramide (Spassieva et al., 2009) have been found to coincide with activation of the UPR. Perturbation to lipid homeostasis activates ER-resident sensors of the UPR, either by lipid disequilibrium or by inducing protein misfolding (reviewed by Volmer and Ron, 2015). Requirement of the EMC for the maturation of key lipid-associated enzymes would have consequences on multiple levels. Decreased transcript levels of lipid metabolic enzymes and induction of ATF4 observed in the alveolar EMC3^−/−^ cell model suggest transcriptional suppression of *de novo* enzyme biosynthesis (Tang et al., 2017). What triggers UPR activation remains undetermined; however, it could be due to activation of the UPR sensors IRE1 (officially known as ERN1) and ATF6 in response to altered lipid metabolism alone (Halbleib et al., 2017; Tam et al., 2018) or in combination with accumulating misfolded membrane proteins.

In yeast, a genetic screen for ER-mitochondria phospholipid transfer identified EMC subunits as factors that promote trafficking of phosphatidylserine (PtdSer) and phosphatidylethanolamine (PtdEtn) between the organelles (Lahiri et al., 2014). Cells lacking EMC1, EMC3, EMC5 and EMC6 had reduced levels of PtdSer and PtdEtn in mitochondria, while overall cellular PtdSer levels were unchanged (Lahiri et al., 2014). This was attributed to reduced transfer of PtdSer from the ER to mitochondria, where PtdSer is decarboxylated to PtdEtn and shuttled back to the ER. In mammalian cells, the EMC appears proximal to SLC25A46, an outer membrane protein and solute carrier involved in lipid exchange at ER-mitochondrial contact sites (Janer et al., 2016). Yet, recent mechanistic insights have not firmly established which EMC clients are the main contributor(s) to ER-mitochondrial contact site physiology. In yeast, aggravating genetic interactions were reported between *EMC6* and genes encoding proteins involved in the metabolism of phospholipids beyond PtdSer and PtdEtn (Lahiri et al., 2014). Among those were: SCS2, which regulates levels of phosphatidylinositol 4-phosphate; the transcription factors INO2 and INO4, which regulate de-repression of inositol-and choline-regulated genes in phospholipid synthesis; SUR1, which is involved in sphingolipid biosynthesis; BTS1, a geranylgeranyl diphosphate synthase; as well as genes encoding the mitochondrial enzymes 3-hydroxyacyl-thioester dehydratase type 2 (HTD2) and the putative malonyl-CoA:ACP transferase (MCT1). Mechanistic details and physiological significance of these associations are unknown but there is the implication of broad pleiotropic lipid-related responsibilities for the EMC in yeast.

### The EMC and sterol homeostasis

Maintenance of sterol homeostasis involves dynamic sensing and response mechanisms located throughout eukaryotic cells, but those concentrated in the ER and endomembrane system responsively control local and global levels of free cholesterol. Recently, the robustness of this homeostatic mechanism was shown to be dependent on the EMC, through insertion of two key enzymes responsible for sterol biosynthesis and storage (Volkmar et al., 2019). The TA protein squalene synthase (FDFT1, hereafter referred to as SQS) catalyses the commitment step of sterol production, irreversibly converting the farnesyl-pyrophosphate (FPP) precursor to squalene and removing it from an alternative fate along the isoprenoid pathway. SQS contains a weakly hydrophobic TMD that requires the EMC for optimal insertion, localisation and, consequently, its activity. Sterol O-acyltransferase 1 (SOAT1) is a multi-pass, ER-resident homo-oligomeric enzyme that esterifies free cellular cholesterol through covalent modification with free fatty acids to generate cholesterol ester, the neutral lipid stored in lipid droplets. By facilitating their biogenesis, the EMC effectively determines the boundaries of viability in response to changes in cholesterol levels. Without the EMC, the narrower window becomes evident when extracellular cholesterol is absent or in surplus, as either condition leads to cell death (Volkmar et al., 2019).

Although it has been demonstrated in autonomous cell culture systems, how exactly EMC dysfunction affects tissues or organismal regulation of cholesterol is not yet clear. As both cholesterol and its steroid hormone derivatives play essential roles in membrane integrity as well as signalling, the EMC is likely to influence both these pathways. Moreover, enabling accurate SQS insertion and, hence, activity has indirect consequences for isoprenoid production, post-translational modifications (e.g. dolichol) and their downstream products. To what degree tissues or organs rich in cholesterol (e.g. liver) or cholesterol esters (e.g. myelin) depend on the EMC remains to be determined. In summary, the diverse metabolic changes in lipid homeostasis exhibited by EMC-deficient cells, indicates the EMC plays an important role in optimising the biogenesis and/or the localisation of lipid-modulating enzymes and transporters.

### The EMC as a viral host factor

Viruses have evolved unique strategies that exploit host biosynthesis and trafficking machinery to promote their own replication. Unbiased, genome-wide forward genetic screens have identified host factors that include signal recognition particles (SRPs), the Sec61 translocon, translocon-associated protein (TRAP), OST complexes as well as ERAD components (Krishnan et al., 2008; Marceau et al., 2016; Saeed et al., 2011; Zhang et al., 2016). This strategy has also implicated the EMC in pathogenicity of flaviviruses, such as West Nile virus (WNV), yellow fever virus (YFV), dengue virus (DENV) and Zika virus (ZV) (Ma et al., 2015; Marceau et al., 2016; Savidis et al., 2016). Loss of EMC2 or EMC3 conferred resistance to WNV-induced cell death, with cells exhibiting a mild defect in WNV replication, as well as increased cell survival (Ma et al., 2015). Even though other EMC subunits were not investigated, the position of EMC2 and EMC3 as ‘core’ subunits required for EMC assembly can reasonably be extrapolated to reflect involvement of the mature EMC. Other studies that implicate different EMC subunits in replication of DENV, ZV and YFV (Le Sommer et al., 2012; Marceau et al., 2016; Ngo et al., 2019; Savidis et al., 2016) may be viewed in the same manner.

Abnormalities appearing at early stages of DENV replication have been described in EMC-deficient cell lines. Although these defects were first attributed to impaired virus entry (Savidis et al., 2016), more recent reports demonstrate aberrant biogenesis and proteasome-mediated degradation of the non-structural polytopic DENV proteins NS4A and NS4B – an observation consistent with the insertase function of the EMC (Lin et al., 2019; Ngo et al., 2019). However, given the complexity of viral entry and replication, it might be premature to rule out a role for the EMC in the accurate biogenesis of cell-surface proteins that mediate viral entry. In this coordinated view, the EMC may be supervising biogenesis of both key host and viral proteins, to concomitantly support entry and replication. Importantly, disruption to the EMC had no effect on hepatitis C virus (HCV) replication, indicating the participation of the EMC is specific and not that of a general factor, involved in protein biogenesis and trafficking (Marceau et al., 2016; Ngo et al., 2019). How the EMC promotes flavivirus replication is only just emerging, with evidence for multiple, not necessarily mutually exclusive, mechanisms being brought forward.

EMC proteins are also harnessed by simian virus 40 (SV40), the archetypic polyomavirus. The SV40 viral particle consists of a non-enveloped capsid that shields the genetic information of the virus. Following receptor-mediated endocytosis, the SV40 virion traffics from endolysosomes to the ER, where it breaches the ER membrane and translocates into the nucleus (reviewed in Toscano and de Haan, 2018). How the ER-to-cytoplasmic transition occurs mechanistically still remains largely enigmatic, but it does depend on ER-resident J proteins (DNAJB12, DNAJB14, DNAJC18), the cytosolic Hsc70-SGTA-Hsp105 chaperone complex, as well as EMC1 (Bagchi et al., 2016). Under this paradigm, EMC1 appears to act as a molecular chaperone; it binds to DNAJC18 and the membrane-embedded SV40 particle to stabilise it, promotes ER-membrane penetration, and prevents premature disassembly (Bagchi et al., 2016). Remarkably, this process is completely dependent on a single, evolutionarily conserved residue (D961) within the predicted TMD of EMC1, possibly enabling the EMC to recognise positively charged regions of the virion (Bagchi et al., 2016). Whether the extensive lumenal domain of EMC1 and its PQQ domain also contribute to virion ER-to-cytosol transport, remains to be determined. In summary, the EMC is part of a growing list of essential host factors that contribute to pathogenic processes, serving as a key biogenesis factor for both endogenous and viral client proteins.

### Moonlighting EMC subunits

Almost all EMC subunits favour assembled rather than an orphaned status (Volkmar et al., 2019), but a variant of EMC10 might be ‘moonlighting’ beyond the primary activity derived from the assembled EMC. EMC10 pleiotropy was first hinted at by the discovery that hematopoietic stem cells express two splice variants. In constrast to full-length TMD-containing EMC10, EMC10-2 (also known as HSS1) lacks a discernible TMD and is secreted into the extracellular space (Junes-Gill et al., 2011; Junes-Gill et al., 2014). EMC10-2 is biologically active and mediates a range of cell-line-dependent effects. Ectopic EMC10-2 expression inhibits growth, alters morphology and exerts global changes on transcription in malignant glioma cells (Junes-Gill et al., 2011). EMC10-2 is a potent angiogenic growth factor for endothelial cells in the post-infarct murine heart (Reboll et al., 2017). Activation of the small GTPases RAC1 and CDC42 along with downstream p38 mitogen-activated kinases (MAPKs) and the p21-activated kinase 2 (PAK2) by ectopic EMC10-2 could indicate cytokine activity through a cell-surface receptor for secreted EMC10. RAC1 and CDC42 enable directed cell migration (Ridley, 2011), which could explain the observed pro-angiogenic activity of EMC10 in myocardial infarction models (Reboll et al., 2017). In one scenario, macrophages that secrete EMC10-2 at ischemic sites would promote angiogenesis and myocardial tissue regeneration. Notably, overexpression of membrane-bound EMC10 in HUVECs promotes endothelial cell proliferation and exerted comparable pro-angiogenic effects (Reboll et al., 2017). How this observation fits into the proposed model of EMC10-2 activity remains to be seen. These roles of EMC10 occur independently of the fundamental EMC function because homozygous EMC10-deficient mice present only mild phenotypes (Diamantopoulou et al., 2017; Probert et al., 2015; Reboll et al., 2017), whereas homozygous mouse knockouts of EMC3, EMC4, EMC6, EMC7 and EMC8 are all lethal (Dickinson et al., 2016).

## Conclusions and perspectives

The EMC has emerged as an important cog of the eukaryotic machinery handling membrane protein biogenesis, where it inserts suboptimal TMDs and improves topological accuracy. Diverse phenotypic outcomes arising from its absence imply that EMC clients underlie a vast range of cellular processes. The EMC is firmly established as both a TA and SAP insertase, and polytopic TMD chaperone at the ER (Chitwood et al., 2018; Guna et al., 2018; Shurtleff et al., 2018). But with these different modes of TMD insertion, many questions remain. How and where the EMC recognises and accepts TMDs is not yet known. To understand how selectivity is conferred will require the structural and physicochemical features of TMDs favoured by the EMC to be determined. Of equal interest will be to define how individual EMC subunits contribute to assembly and the insertase/chaperone activity. At present, we lack the high-resolution structural detail of the EMC required to establish the presence of a TMD-accepting hydrophobic groove or channel. For this, high resolution Cryo-EM structures of intact, native EMCs – ideally in complex with client TMDs – will be required, and such endeavours are most certainly underway.

Many questions surrounding client accessibility to the EMC still abound, and a key one to answer is the mechanism and specificity of TA protein transfer or handoff from CaM to the EMC. The EMC can autonomously promote TA insertion into liposomes (Guna et al., 2018), but the determinants of Sec61-autonomous versus Sec61-cooperative insertase activity are less clear. Current models of EMC–Sec61 coordination for polytopic clients favour spatial proximity and transient interactions between complexes. Yet, how EMC clients (e.g. GPCRs) signal to the Sec61 translocon in response to SAP insertion and how both complexes coordinate to insert polytopic proteins are yet to be experimentally demonstrated. In both cases, a mechanism for the EMC to release clients into the lipid bilayer, akin to the lateral gate of Sec61, is required to complete the picture of how targeting and insertion cycles operate. This is also relevant when we consider that the EMC may also routinely engage internal TMDs from polytopic membrane proteins.

Deficient cell lines and genetically modified model organisms used in conjunction with unbiased, quantitative proteomic approaches and ribosome profiling, have allowed the breadth of potential EMC clients within the proteome to be appreciated. However, cognate EMC clients and those proteins impacting indirectly must be fully differentiated in order to understand the rules that govern EMC selectivity. Accomplishing this requires complementary biochemical assays that are capable of distinguishing primary from secondary activities. There are a number of examples of EMC clients that exhibit tissue-specific expression patterns (e.g. rhodopsins). The tissue-specific phenotypes apparent with EMC loss might be attributable to as yet unknown TM proteins with restricted expression. Identifying the principal EMC clients present within different tissues will provide greater insight into its influence on physiological functions (Fig. 2B).

Furthermore, any direct involvement of the EMC in the aetiology of human disease is only beginning to be uncovered. Recent technological advancements enabling genome-wide forward genetic screening approaches revealed the EMC to be a key host factor for flavivirus propagation. Exome sequencing correlated subunit point mutations to severe neurodegenerative syndromes as well as schizophrenia. How and why naturally occurring somatic and inherited mutations in EMC subunits or their clients promote disease development and progression is still poorly understood. However, by understanding the fundamental responsibilities of the EMC, work can now begin to tease apart the complex phenotypes by looking for dysregulation of key clients in affected cell types and the consequences they elicit. Owing to the complexity of reported disease-related EMC phenotypes, efforts to isolate underlying defects have already begun but will require further comprehensive multi-omics as well as targeted biochemical approaches in appropriate model systems.

Taken together, these recent advances in our understanding of the EMC have opened the door to an exciting new set of questions. Future studies will not only aim to clarify the mechanism, selectivity and client proteins of the EMC but will also expand our understanding of its role in fundamental processes of protein biogenesis that establishes tissue homeostasis and organismal viability.
